# Lung Function Trajectory Using Race-Specific vs Race-Neutral Global Lung Function Initiative Coefficients

**DOI:** 10.1001/jamanetworkopen.2025.7304

**Published:** 2025-04-25

**Authors:** Darshali A. Vyas, Sophia Zhao, Peggy S. Lai, Josanna Rodriguez-Lopez, Eric P. Schmidt, Robert Brown, Kathryn A. Hibbert, Charles C. Hardin, Crystal M. North

**Affiliations:** 1 Division of Pulmonary and Critical Care Medicine, Massachusetts General Hospital, Boston; 2 Medical Practice Evaluation Center, Massachusetts General Hospital, Boston

## Abstract

**Importance:**

The use of race-based coefficients in pulmonary function testing has led to the recent development of the race-neutral Global Lung Function Initiative (GLI) reference equation (hereafter GLI Global). The performance of GLI Global in comparison to race-adjusted coefficients (hereafter GLI 2012) has not been well characterized.

**Objective:**

To compare the implications of GLI 2012 vs GLI Global reference equations for lung function trajectory.

**Design, Setting, and Participants:**

This cohort study at Massachusetts General Hospital analyzed data of patients aged 18 to 95 years who completed spirometry testing between January 1, 1997, and December 31, 2020. Data analysis was performed from January 2023 to November 2024.

**Exposures:**

GLI Global and GLI 2012 reference equations to define lung function.

**Main Outcomes and Measures:**

Proportion of patients with recategorized lung function (FEV_1_ and FVC) based on the 2 reference equations and their lung function trajectory over time. Lung function metrics included forced expiratory volume in the first second of expiration (FEV_1_), forced vital capacity (FVC), and FEV_1_ to FVC ratio. The *z* scores for FEV_1_ and FVC were calculated using the GLI 2012 and GLI Global reference equations, with a score lower than −1.64 considered abnormal. Patients were categorized into 1 of 4 groups based on their *z* scores: normal to normal (normal *z* score on both equations); abnormal to normal (abnormal *z* score based on GLI 2012 equations but normal based on the GLI Global equation); normal to abnormal (normal *z* score on GLI 2012 equations but abnormal on the GLI Global equation); and abnormal to abnormal (abnormal *z* score on both equations).

**Results:**

The sample included a total of 24 662 patients (988 Black [4.0%] and 22 297 White [90.4%] patients; 13 108 women [53.2%]) with a mean (SD) age of 57.6 (15.7) years who completed a median (IQR) of 3.0 (2.0-5.0) sets of spirometry over a median (IQR) of 2.6 (0.8-6.6) years between 1997 and 2020. Among Black patients, 190 (19.2%) had either their FEV_1_ or FVC recategorized from normal to abnormal using the GLI Global reference equation. The subset of Black patients whose lung function was recategorized from normal to abnormal exhibited FEV_1_ decline (−2.06%; 95% CI, −3.47% to −0.64%; *P* = .56) that was similar to decline in Black patients whose lung function was characterized as abnormal (−1.89%; 95% CI, −2.58% to −1.19%; *P* = .84) using both the GLI Global and GLI 2012 reference equations. Among White patients, 3348 (15.0%) had either their FEV_1_ or FVC recategorized from abnormal to normal using the GLI Global equation. FEV_1_ decline in these patients (−1.82%; 95% CI, −2.55% to −1.08%; *P* = .70) was similar to the decline in White patients with normal spirometry (−1.97%; 95% CI, −2.26% to −1.69%; *P* = .70) regardless of the reference equation used. Patterns in FVC trajectory among both Black and White participants were not consistent among participants whose spirometry was recategorized between normal and abnormal compared with those whose spirometry remained normal or remained abnormal regardless of the reference equation used.

**Conclusions and Relevance:**

This cohort study found that Black patients whose lung function was recategorized from normal to abnormal using the GLI Global reference equation exhibited FEV_1_ decline similar to that in Black patients whose lung function was classified as abnormal regardless of which equation was used. This finding suggests that a race-neutral approach to spirometry interpretation may allow a more accurate identification of lung pathology in Black patients.

## Key Points

**Question:**

With use of a race-neutral Global Lung Function Initiative (GLI) global coefficient in spirometry interpretation, do patients whose lung function was recategorized between normal and abnormal have accelerated lung function decline over time?

**Findings:**

In this cohort study of 24 662 patients, Black patients whose lung function was recategorized from normal to abnormal using the GLI Global reference equation had accelerated lung function decline over time, while White patients with recategorized lung function of abnormal to normal did not have accelerated decline over time. Lung function decline was similar in Black patients with recategorized lung function and Black patients whose lung function was abnormal using race-neutral and race-adjusted reference equations.

**Meaning:**

Application of the GLI Global coefficient in spirometry interpretation may allow increased detection of abnormal lung function among Black patients.

## Teaser

This cohort study compares the performance of the Global Lung Function Initiative (GLI) race-neutral reference equation and the GLI race-adjusted equation for interpreting pulmonary function testing values in Black and White patients.

## Introduction

The invention of the spirometer in the 19th century was motivated by racist desires to substantiate notions of Black inferiority; since then, race has remained intimately tied to the interpretation of lung function.^1,2^ More recently, there have been attempts to revise clinical tools that inappropriately adjusted for race and may exacerbate racial inequity.^3,4,5,6,7^ In pulmonary medicine, this revision has manifested in a reconsideration of the use of race-specific reference equations in the interpretation of spirometry.^8,9^

Lower lung function is associated with higher mortality independent of race, such that low lung function among patients of different racial and/or ethnic backgrounds has similar implications for mortality.^10,11,12^ Furthermore, the use of race-adjusted reference equations compared with race-neutral reference equations does not improve prediction of clinical events^13^ or postsurgical complications^14^ and indeed may lead to underestimation of the severity of chronic obstructive pulmonary disease and underrecognition of emphysema among Black patients.^15,16^ As a result, in April 2023, the American Thoracic Society (ATS) updated its official guidance on spirometry interpretation.^17^ Specifically, the ATS guidelines recommend replacing the 2012 Global Lung Function Initiative (GLI) race-adjusted reference equations^18^ (hereafter GLI 2012, in which separate equations are applied to patients of Black, North Asian, Southeast Asian, White, and other race and ethnicity) with the new GLI Global race-neutral reference equation.^19^ Thus, in the US, GLI Global is the only reference equation that should be applied to all patients regardless of race and ethnicity.

The use of the race-neutral GLI Global reference equation will result in a proportion of patients from racial and ethnic minoritized groups being recategorized from so-called normal to abnormal lung function as well as a proportion of White patients being recategorized from abnormal to normal lung function.^20,21^ However, questions remain as to the diagnostic accuracy of these recategorizations,^22^ and data on the implications of the GLI Global reference equation in clinical settings are lacking.^23,24^ To better understand the diagnostic accuracy of these recategorizations, we retrospectively applied both race-adjusted GLI 2012 and race-neutral GLI Global reference equations to spirometry tests from a cohort of patients in a large academic medical center. Specifically, we compared the implications of these equations for lung function trajectory between patients whose percent predicted lung function was recategorized from normal to abnormal using the GLI Global reference equation and those whose percent predicted lung function categorization remained unchanged regardless of the equation applied.

## Methods

This retrospective cohort study included patients between 18 and 95 years of age who completed spirometry at Massachusetts General Hospital between January 1, 1997, and December 31, 2020. The Mass General Brigham Institutional Review Board approved the study and waived the informed consent requirement given the retrospective nature of the analysis. We followed the Strengthening the Reporting of Observational Studies in Epidemiology (STROBE) reporting guideline.

We abstracted patient demographics from spirometry reports, which included age, height, weight, and smoking history. Self-identified patient race and ethnicity were recorded as entered in the electronic medical record (EMR). Smoking history was self-reported by patients and categorized as current, former, or never smoker. Missing demographic data or demographic data that were discrepant between repeated spirometry of the same patient were reconciled by EMR review. Spirometry was performed using dry rolling seal spirometers (Medisoft) and ComPAS software (Morgan Scientific). We removed spirometry that did not meet ATS acceptability or reproducibility criteria,^25,26,27^ patients with only 1 spirometry test during the study period, and patients older than 95 years because GLI equation values cannot be calculated for individuals older than 95 years. We also removed Asian patients because GLI equations require the ability to differentiate between patients of Southeast Asian and Northeast Asian descent, but this level of granularity is not available for Asian patients in the EMR, and patients for whom race and ethnicity were not recorded in the EMR. Lung function metrics extracted from spirometry reports included prebronchodilator forced expiratory volume in the first second of expiration (FEV_1_), forced vital capacity (FVC), and FEV_1_ to FVC ratio.

We calculated the *z* scores for FEV_1_ and FVC using the race-specific GLI 2012 coefficients^19^ (for patients of Black, White, and other race and ethnicity) and then compared them with the *z* scores using the GLI Global reference equation.^20^ We defined a *z* score of less than −1.64 (below the fifth percentile) as abnormal.^18^ Using the first spirometry report during the study period, we categorized patients into 1 of 4 groups, separately for their FEV_1_ and FVC, based on their *z* scores: a normal *z* score by both the GLI 2012 and the GLI Global equations (labeled normal to normal), an abnormal *z* score based on GLI 2012 equations but normal by the GLI Global equation (labeled abnormal to normal), a normal *z* score by GLI 2012 equations but abnormal by the GLI Global equation (labeled normal to abnormal), and an abnormal *z* score by both the GLI 2012 and the GLI Global equations (labeled abnormal to abnormal). As a result, each patient was assigned to 1 of the 4 lung function recategorization populations for their baseline FEV_1_ and baseline FVC, separately (eFigure 1 in Supplement 1).

### Statistical Analysis

We described demographic characteristics using descriptive statistics, as appropriate. We then quantified the proportion of Black patients and White patients whose FEV_1_ or FVC were recategorized between normal and abnormal when the GLI 2012 reference equations were replaced with the GLI Global reference equation, and proportion differences were compared using the McNemar test. Next, we compared longitudinal change in FEV_1_ and FVC, represented as the percent change from baseline values using raw FEV_1_ and FVC (in liters), among the 4 groups of patients using linear mixed-effects models with random intercepts and random slopes, accounting for repeated observations within each participant.^28,29^ We defined baseline values as the first spirometry report in the database and identified the reference population in the regression models as the population for whom spirometry remained categorized as normal regardless of which reference equation was used (the normal to normal category). Regression coefficients for the change in lung function were obtained from multivariate models that were adjusted for potential confounders, which were chosen a priori based on established associations with lung function and included age, sex, and height.

We defined statistical significance as a nominal α level of .05, and all reported *P* values were 2-sided. No adjustment was performed for multiple comparisons. Statistical analysis was performed from January 2023 to November 2024 using R, version 4.1.2 (R Project for Statistical Computing).

## Results

A total of 24 662 patients (13 108 women [53.2%] and 11 554 men [46.8%]) with a mean age of 57.6 (15.7) years completed a median (IQR) of 3.0 (2.0-5.0) sets of spirometry between 1997 and 2020. Of these patients, 591 (2.4%) had Asian, 988 (4.0%) had Black, 22 297 (90.4%) had White, 452 (1.8%) had other, and 334 (1.4%) had unknown race and ethnicity. The median (IQR) per-participant follow-up duration was 2.6 (0.8-6.6) years, and the median (IQR) time interval between spirometry was 4.9 (2.0-12.0) months. (Table 1; eFigures 2 and 3 and eTable 1 in Supplement 1). A small percentage of participants (6.5% [1615]) were current smokers at the time of spirometry, while 30.4% (7501) were former smokers, 28.3% (6971) were never smokers, and 34.8% (8575) had unknown smoking history. Patients who had ever smoked had a median (IQR) exposure of 30.8 (12.0-57.0) pack-years.

**Table 1.** Baseline Characteristics of Patients

Characteristic	Patients, No. (%)		
	Total cohort (N = 24 662)	White race (n = 22 297)	Black race (n = 988)
Age, mean (SD), y	57.6 (15.7)	58.1 (15.6)	52.3 (14.8)
Sex			
Women	13 108 (53.2)	11 821 (53.0)	566 (57.3)
Men	11 554 (46.8)	10 476 (47.0)	422 (42.7)
Race and ethnicity^a^			
Asian^b^	591 (2.4)	NA	NA
Black	988 (4.0)	NA	NA
White	22 297 (90.4)	NA	NA
Other^b^	452 (1.8)	NA	NA
Unknown	334 (1.4)	NA	NA
Smoking status			
Former smoker	7501 (30.4)	6889 (30.9)	208 (21.1)
Never smoker	6971 (28.3)	5840 (26.2)	364 (36.8)
Current smoker	1615 (6.5)	1434 (6.4)	86 (8.7)
Unknown	8575 (34.8)	8134 (36.5)	330 (33.4)
Pack-years, median (IQR), No.^c^	30.8 (12.0-57.0)	31.5 (12.0-58.0)	24.0 (9.0-43.0)
Height, mean (SD), m	1.7 (0.1)	1.7 (0.1)	1.7 (0.1)
Weight, mean (SD), kg	80.2 (20.8)	80.4 (20.7)	86.3 (22.7)
BMI, mean (SD)^d^	28.5 (6.8)	28.6 (6.8)	30.6 (7.9)
BMI categories			
Underweight: <18.5	649 (2.7)	568 (2.6)	23 (2.4)
Normal weight: 18.5 to <25.0	7075 (29.3)	6339 (29.1)	219 (22.6)
Overweight: 25.00 to <30.0	7995 (33.1)	7236 (33.2)	281 (28.9)
Obese: ≥30.0	8426 (34.9)	7654 (35.1)	448 (46.1)
Follow-up time, median (IQR), y	2.6 (0.8-6.6)	2.7 (0.8-6.7)	3.0 (0.8-7.4)
No. of PFTs, median (IQR)	3.0 (2.0-5.0)	3.0 (2.0-5.0)	3.0 (2.0-5.0)

Abbreviations: BMI, body mass index (calculated as weight in kilograms divided by height in meters squared); NA, not applicable; PFT, pulmonary function test.

^a^ Race and ethnicity were self-identified and obtained from the electronic medical record.

^b^ Asian included Northeast Asian and Southeast Asian. Other included American Indian or Alaska Native, Native Hawaiian or Other Pacific Islander, and other.

^c^ Among current or former smokers.

^d^ BMI data were missing for 517 participants due to missing weight data.

Among the 988 Black patients, 110 (11.1%) had their FEV_1_ recategorized from normal to abnormal, 111 (11.2%) had their FVC recategorized from normal to abnormal, and 190 (19.2%) had either FEV_1_ or FVC recategorized from normal to abnormal (Table 2 and Table 3). Among the 22 297 White patients, 1808 (8.1%) had their FEV_1_ recategorized from abnormal to normal, 2097 (9.4%) had their FVC recategorized from abnormal to normal, and 3348 (15.0%) had either FEV_1_ or FVC recategorized from abnormal to normal (Table 2 and Table 3). In terms of baseline demographic characteristics, Black patients whose lung function was recategorized from normal to abnormal were less commonly women compared with Black patients whose lung function was not recategorized (87 of 190 [45.8%] vs 479 of 798 [60.0%]; *P* < .001), while White patients whose lung function was recategorized had statistical but not substantive differences in their demographic characteristics (Table 4). Paradoxically, FEV_1_ was recategorized from normal to abnormal among an additional 15 White patients (0.1%), and FVC was recategorized from normal to abnormal among an additional 7 White patients (0%) (Table 3). These 22 White patients whose FEV_1_ and FVC were paradoxically recategorized from normal to abnormal were nearly all men (19 [86.4%]) with a mean (SD) age of 87.8 (4.3) years and mean (SD) height of 1.7 (0.1) meters (eTable 2 in Supplement 1).

**Table 2.** Proportion of Patients Whose Lung Function Interpretation Was Recategorized With the Shift From GLI 2012 to GLI Global Reference Equations^a^

Pulmonary function test and race	Patients, No. (%)			
	Normal to normal	Normal to abnormal	Abnormal to normal	Abnormal to abnormal
Black race (n = 988)				
FEV_1_	456 (46.2)	110 (11.1)	0	422 (42.7)
FVC	512 (51.8)	111 (11.2)	0	365 (36.9)
White race (n = 22 297)				
FEV_1_	12 840 (57.6)	15 (0.1)	1808 (8.1)	7634 (34.2)
FVC	14 860 (66.7)	7 (0.0)	2097 (9.4)	5333 (23.9)

Abbreviations: FEV_1_, forced expiratory volume in the first second of expiration; FVC, forced vital capacity; GLI, Global Lung Function Initiative.

^a^ The first categorization (normal or abnormal) designates how lung function was classified using GLI 2012 spirometry reference equations, and the second categorization (normal or abnormal) designates GLI Global application. For example, normal to abnormal indicates the individual had normal lung function under GLI 2012 reference equations but was recategorized as abnormal when the GLI Global reference equation was applied.

**Table 3.** Proportion of Patients Whose Lung Function Was Recategorized as Normal and Abnormal With the Shift From GLI 2012 to GLI Global Reference Equations^a^

Pulmonary function test	Patients, No. (%)		
	Normal to abnormal		Abnormal to normal
	Black race (n = 988)	White race (n = 22 297)	White race (n = 22 297)^b^
FVC	111 (11.2)	7 (0.0)	2097 (9.4)
FEV_1_	110 (11.1)	15 (0.1)	1808 (8.1)
FVC or FEV_1_	190 (19.2)	22 (0.1)	3348 (15.0)

Abbreviations: FEV_1_, forced expiratory volume in the first second of expiration; FVC, forced vital capacity; GLI, Global Lung Function Initiative.

^a^ The first categorization (normal or abnormal) designates how lung function was classified using GLI 2012 spirometry reference equations, and the second categorization (normal or abnormal) designates GLI Global application.

^b^ There were no Black patients whose FEV_1_ or FVC were recategorized from abnormal to normal.

**Table 4.** Demographic Characteristics of Patients Whose FEV_1_ or FVC Was Recategorized or Not Recategorized With the Application of the GLI Global Reference Equation

Characteristic	Black patients, No. (%) (n = 988)			White patients, No. (%) (n = 22 275)^a^		
	Recategorized (n = 190)	Not recategorized (n = 798)	*P* value	Recategorized (n = 3348)	Not recategorized (n = 18 927)	*P* value
Age, mean (SD), y	53.2 (15.9)	52.0 (14.5)	.34	55.0 (13.9)	58.57 (15.8)	<.001
Women	87 (45.8)	479 (60.0)	<.001	1937 (57.9)	9881 (52.2)	<.001
Smoking status						
Previous smoker	48 (25.3)	160 (20.1)	.15	845 (25.2)	6031 (31.9)	<.001
Never smoker	58 (30.5)	306 (38.3)		736 (22.0)	5098 (26.9)	
Current smoker	15 (7.9)	71 (8.9)		297 (8.9)	1136 (6.0)	
Unknown	69 (36.3)	261 (32.7)		1470 (43.9)	6662 (35.2)	
Pack-years, median (IQR)^b^	22.0 (9.1-39.8)	24.5 (8.2-43.3)	.69	38.5 (17.0-64.5)	30.0 (11.7-57.0)	<.001
Height, mean (SD), m	1.7 (0.1)	1.7 (0.1)	.001	1.7 (0.1)	1.7 (0.1)	<.001
Follow-up time, median (IQR), y	3.3 (0.8-7.8)	3.0 (0.8-7.3)	.64	3.1 (0.9-7.9)	2.6 (0.8-6.5)	<.001
No. of PFTs, median (IQR)	3.0 (2.0-4.0)	3.0 (2.0-5.0)	.43	3.0 (2.0-6.0)	3.0 (2.0-5.0)	<.001
Baseline FVC, mean (SD), L	2.8 (0.8)	2.7 (1.1)	.39	2.9 (0.8)	3.2 (1.2)	<.001
Baseline FEV_1_, mean (SD), L	2.1 (0.6)	2.1 (0.9)	.94	2.1 (0.7)	2.4 (1.0)	<.001

Abbreviations: FEV_1_, forced expiratory volume in the first second of expiration; FVC, forced vital capacity; GLI, Global Lung Function Initiative; PFT, pulmonary function test.

^a^ The 22 White patients whose lung function was paradoxically recategorized from normal to abnormal are excluded from this table. Their characteristics are specified in eTable 2 in Supplement 1.

^b^ Among current or former smokers.

Lung function trajectory across recategorization populations varied by race and ethnicity (eTables 3-7 and eFigures 4-7 in Supplement 1). Among Black patients, those whose FEV_1_ was recategorized from normal to abnormal had an annual decline in FEV_1_ from baseline (−2.06%; 95% CI, −3.47% to −0.64%; *P* = .56) that was greater than the annual FEV_1_ decline among those whose FEV_1_ remained normal regardless of the reference equation used (−1.59%; 95% CI, −2.29% to −0.89%; *P* = .56) but was similar among those whose FEV_1_ remained abnormal regardless of the equation used (−1.89%; 95% CI, −2.58% to −1.19%; *P* = .84) (Figure). On the contrary, among White patients, those whose FEV_1_ was recategorized from abnormal to normal had an annual decline in FEV_1_ from baseline (−1.82%; 95% CI, −2.55% to −1.08%; *P* = .70) that was less than that among those whose FEV_1_ remained abnormal regardless of the reference equation used (−2.45%; 95% CI, −2.80% to −2.11%; *P* = .12) but was similar among those whose FEV_1_ remained normal regardless of the equation used (−1.97%; 95% CI, −2.26% to −1.69%; *P* = .70) (Figure). Although differences were not statistically significant, the results suggest that Black patients whose FEV_1_ was recategorized from normal to abnormal may have undetected lung pathology and that White patients whose FEV_1_ was recategorized from abnormal to normal may be correctly reclassified to normal lung function.

**Figure.**
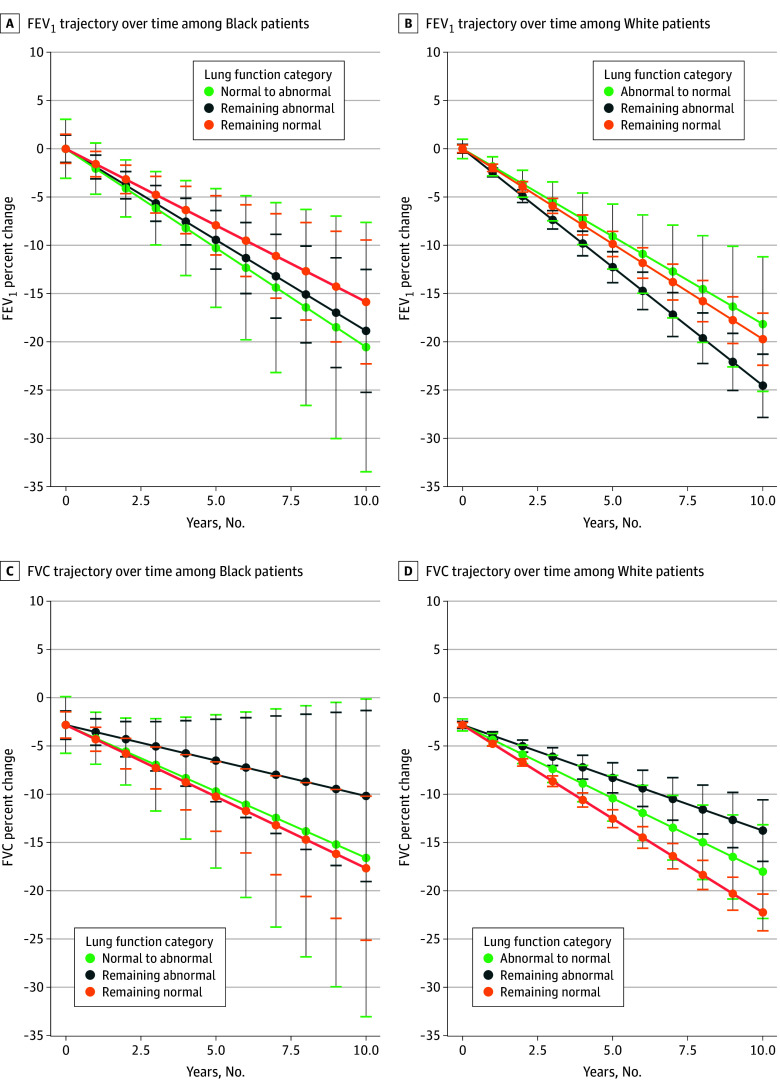
Trajectories of Forced Expiratory Volume in the First Second of Expiration (FEV_1_) and Forced Vital Capacity (FVC) After Application of the Global Lung Function Initiative Global Reference Equation Error bars represent 95% CIs.

Patterns in FVC trajectory were less apparent. Black patients whose FVC was recategorized from normal to abnormal had an annual decline in FVC from baseline (−1.08%; 95% CI, −2.44% to 0.29%) that was similar to the FVC decline in Black patients whose FVC remained normal regardless of the equation used (−1.16%; 95% CI, −1.78% to −0.54%; *P* = .91) (Figure). White patients whose FVC was recategorized from abnormal to normal had an annual decline in FVC from baseline (−1.19%; 95% CI, −1.58% to −0.79%) that was intermediate between White patients whose FVC remained either normal (−1.52%; 95% CI, −1.67% to −1.36%; *P* = .13) or abnormal (−0.85%; 95% CI, −1.11% to −0.60%; *P* = .17) regardless of the equation used (Figure). All FEV_1_ and FVC trajectory findings among Black and White patients remained unchanged in sensitivity analyses, which limited the analytic cohort to patients at least 30 years of age or patients who completed at least 3 or 5 sets of spirometry during the study period (eTables 8-13 in Supplement 1).

## Discussion

In this single-center cohort study, we found that applying the race-neutral GLI Global spirometry reference equation instead of the race-adjusted GLI 2012 reference equations was associated with an increased proportion of Black patients identified as having abnormal lung function and a decreased proportion of White patients identified as having abnormal lung function. The FEV_1_ trajectory among Black patients who were recategorized from normal to abnormal lung function was similar to that of Black patients who had abnormal FEV_1_ regardless of the reference equation used, while the FEV_1_ trajectory among White patients who were recategorized from abnormal to normal lung function was similar to that of White patients who had normal FEV_1_ regardless of the reference equation used. These findings suggest that these recategorizations of lung function among Black and White patients are accurate.

Our confidence in the appropriateness of these recategorizations is critical because the implementation of the GLI Global reference equation carries the burden of redefining which patients are regarded as having normal vs abnormal lung function. Newly reclassifying patients’ lung function as normal or abnormal has far-reaching consequences, from determining screening frequency and access to specialty care to identifying surgical candidacy, employment eligibility, and insurance coverage.^30^ Previous studies have demonstrated that use of race-adjusted tools can lead to the underdiagnosis of lung disease in Black patients, but it remains uncertain whether Black patients whose lung function would be recategorized as abnormal truly have undiagnosed lung disease or whether their lung function would instead be falsely pathologized as abnormal and subject to unnecessary testing and interventions.^15,16,17^ Our findings suggest that this subset of Black patients has been inappropriately assigned to normal lung function because their rate of lung function decline over time is similar to Black patients whose lung function is considered abnormal regardless of the reference equation used.

While clear patterns were apparent for FEV_1_ trajectories across the study population, the FVC trajectories were less defined. Among White patients, the FVC trajectory among those whose lung function was recategorized from abnormal to normal did not appear to behave similarly to the trajectory of patients whose lung function remained either normal or abnormal regardless of the reference equation used. Among Black patients, the FVC trajectory among those whose lung function was recategorized from normal to abnormal appeared to behave similarly to the trajectory of those whose lung function remained normal regardless of the reference equation used, which was unexpected. Given that we lacked data on mortality, we were unable to account for the competing risk of death in this study population. It is likely that those with the worse lung function, as indicated by an abnormal FVC even in the setting of race-adjusted tools, would be at highest risk of mortality and other outcomes. If there is differential loss to follow-up among the sickest patients in the population (those with the lowest FVC), then the FVC trajectory would be most preserved among that cohort of patients, as was the case in both the White and Black patients in this study. This unexpected result underscores the preliminary nature of these findings and highlights the importance of exploring the validity of the results in other cohorts.

Some have expressed concern that the new ATS recommendation to use the race-neutral GLI Global reference equation for spirometry interpretation may result in the underdiagnosis of lung disease among White patients. Fifteen percent of White patients in this study had their lung function recategorized from abnormal to normal when the GLI Global reference equation was applied. However, the FEV_1_ decline in these patients was similar to the FEV_1_ decline in White patients whose lung function remained normal regardless of reference equation, suggesting that White patients who change from an abnormal to normal spirometry interpretation are less likely to have lung pathology that is overlooked by the transition to race-neutral reference equation.

Unexpectedly, a small number of White patients were recategorized from having normal to abnormal lung function with the use of the GLI Global reference equation. These 22 White patients tended to be older and were predominantly men. These patients may simply be at the extremes of age, such that the stability of the equations is less reliable. Alternatively, this population may represent an outlier within the GLI Global model that merits further exploration.

### Strengths and Limitations

The main strength of this study is that it used data from a large database of spirometry tests spanning 24 years. Such data allowed us to add to the published literature by comparing the lung function trajectory across populations whose spirometry interpretation changed after the removal of adjustment for race.

The study also has several limitations. First, we did not distinguish between obstructive, restrictive, and mixed patterns of spirometry abnormalities. Due to limitations in our dataset, we were unable to differentiate between specific clinical diagnoses or indications for obtaining spirometry and did not investigate clinical outcomes, such as hospitalizations. We also did not exclude patients who underwent lung transplant or lung volume reduction surgery, both of which would affect spirometry trajectory by causing sudden improvement in or normalization of lung function, although we expected the number of patients who underwent these procedures to be small compared with the overall size of the study cohort. We chose not to adjust for smoking history because those data were missing for nearly 35% of the patient population, and we were unable to account for mortality as a competing risk due to incomplete availability of mortality data. Additionally, despite the long-term nature of the cohort, patients were followed up for a median duration of only 2.6 years, which may not have been long enough to identify accelerated lung function decline. However, this would also be expected to bias the results toward the null, further emphasizing the importance of the findings. An alternative approach to normalizing lung function is the use of the forced expiratory volume in 1 second quotient (FEV_1_Q) instead of reference equations.^31^ Given our primary objective to compare the implications of both reference equations for lung function trajectory, we elected to follow FEV_1_ over time rather than alternative measures such as FEV_1_Q, which seeks to minimize the errors associated with use of reference equations. Compared with the FEV_1_ trajectory, the FVC values in this cohort followed a less clearly defined pattern, which requires further exploration. Furthermore, the single-center setting and the relatively small number of patients from racial and ethnic minority groups may limit generalizability outside of quaternary health care populations; the retrospective design also limits any causal conclusions.

## Conclusions

The study found that Black patients whose lung function was recategorized from normal to abnormal with the use of the race-neutral GLI Global reference equation had FEV_1_ decline over time that behaved similarly to the trajectory in Black patients whose lung function was categorized as abnormal regardless of which equation was used. This result suggests that moving toward a race-neutral approach to spirometry interpretation may allow a more accurate identification of Black patients with lung disease. Future work is needed to explore these associations among racial and ethnic minority populations, including Asian patients, to assess whether patients whose lung function is recategorized by the GLI Global reference equation have other differences in clinical interventions or outcomes and to identify how alternative spirometry normalization approaches, such as FEV_1_Q, are affected by adopting race-neutral reference equations.

## Notes

Supplement 1. **eFigure 1.** Patient Categorizations **eFigure 2.** Patient Flow Diagram **eFigure 3.** Follow-up Duration Among Patients With Serial Spirometry in Study Cohort **eFigure 4.** Model Performance for Multivariable Regression Model for FEV1 Among Black Individuals in Cohort **eFigure 5.** Model Performance for Multivariable Regression Model for FVC Among Black Individuals in Cohort **eFigure 6.** Model Performance for Multivariable Regression Model for FEV1 Among White Individuals in Cohort **eFigure 7.** Model Performance for Multivariable Regression Model for FVC Among White Individuals in Cohort **eTable 1.** Number and Time Interval of Spirometry Tests Among Patients in the Analytic Cohort **eTable 2.** Characteristics of White Patients Re-Categorized From Normal to Abnormal Lung Function With GLI-Global Prediction Equation **eTable 3.** Multivariable Regression Model Output for FEV1 Among Black Individuals in Cohort **eTable 4.** Multivariable Regression Model Output for FEV1 Among White Individuals in Cohort **eTable 5.** Multivariable Regression Model Output for FVC Among Black Individuals in Cohort **eTable 6.** Multivariable Regression Model Output for FVC Among White Individuals in Cohort **eTable 7.** Comparison of Slopes for Lung Function Trajectory Between Recategorized Groups **eTable 8.** Sensitivity Analysis of FEV1 Trajectory Over Time in Patients With Three or More Spirometry Tests **eTable 9.** Sensitivity Analysis of FVC Trajectory Over Time in Patients With Three or More Spirometry Tests **eTable 10.** Sensitivity Analysis of FEV1 Trajectory Over Time in Patients With Five or More Spirometry Tests **eTable 11.** Sensitivity Analysis of FVC Trajectory Over Time in Patients With Five or More Spirometry Tests **eTable 12.** Sensitivity Analysis of FEV1 Trajectory Over Time in Patients 30 Years or Older Within Cohort **eTable 13.** Sensitivity Analysis of FVC Trajectory Over Time in Patients 30 Years or Older Within Cohort

Supplement 2.
Data Sharing Statement
