# Supplementary material for: Lung Function Trajectory Using Race-Specific vs Race-Neutral Global Lung Function Initiative Coefficients
Source: JAMA Netw Open. 2025 Apr 25;8(4):e257304. doi: 10.1001/jamanetworkopen.2025.7304 (PMC12032560; doi:10.1001/jamanetworkopen.2025.7304)
Supplement: Supplement 1. — eFigure 1. Patient Categorizations eFigure 2. Patient Flow Diagram eFigure 3. Follow-up Duration Among Patients With Serial Spirometry in Study Cohort eFigure 4. Model Performance for Multivariable Regression Model for FEV1 Among Black Individuals in Cohort eFigure 5. Model Performance for Multivariable Regression Model for FVC Among Black Individuals in Cohort eFigure 6. Model Performance for Multivariable Regression Model for FEV1 Among White Individuals in Cohort eFigure 7. Model Performance for Multivariable Regression Model for FVC Among White Individuals in Cohort eTable 1. Number and Time Interval of Spirometry Tests Among Patients in the Analytic Cohort eTable 2. Characteristics of White Patients Re-Categorized From Normal to Abnormal Lung Function With GLI-Global Prediction Equation eTable 3. Multivariable Regression Model Output for FEV1 Among Black Individuals in Cohort eTable 4. Multivariable Regression Model Output for FEV1 Among White Individuals in Cohort eTable 5. Multivariable Regression Model Output for FVC Among Black Individuals in Cohort eTable 6. Multivariable Regression Model Output for FVC Among White Individuals in Cohort eTable 7. Comparison of Slopes for Lung Function Trajectory Between Recategorized Groups eTable 8. Sensitivity Analysis of FEV1 Trajectory Over Time in Patients With Three or More Spirometry Tests eTable 9. Sensitivity Analysis of FVC Trajectory Over Time in Patients With Three or More Spirometry Tests eTable 10. Sensitivity Analysis of FEV1 Trajectory Over Time in Patients With Five or More Spirometry Tests eTable 11. Sensitivity Analysis of FVC Trajectory Over Time in Patients With Five or More Spirometry Tests eTable 12. Sensitivity Analysis of FEV1 Trajectory Over Time in Patients 30 Years or Older Within Cohort eTable 13. Sensitivity Analysis of FVC Trajectory Over Time in Patients 30 Years or Older Within Cohort [file jamanetwopen-e257304-s001.pdf]

Supplemental Online Content

Vyas DA, Zhao S, Lai PS, et al. Lung function trajectory using race-specific vs race-neutral Global Lung Function Initiative coefficients. *JAMA Netw Open*. 2025;8(4);e257304. doi:10.1001/jamanetworkopen.2025.7304

eFigure 1. Patient Categorizations
eFigure 2. Patient Flow Diagram
eFigure 3. Follow-up Duration Among Patients With Serial Spirometry in Study Cohort
eFigure 4. Model Performance for Multivariable Regression Model for FEV1 Among Black Individuals in Cohort
eFigure 5. Model Performance for Multivariable Regression Model for FVC Among Black Individuals in Cohort
eFigure 6. Model Performance for Multivariable Regression Model for FEV1 Among White Individuals in Cohort
eFigure 7. Model Performance for Multivariable Regression Model for FVC Among White Individuals in Cohort
eTable 1. Number and Time Interval of Spirometry Tests Among Patients in the Analytic Cohort
eTable 2. Characteristics of White Patients Re-Categorized From Normal to Abnormal Lung Function With GLI-Global Prediction Equation
eTable 3. Multivariable Regression Model Output for FEV1 Among Black Individuals in Cohort
eTable 4. Multivariable Regression Model Output for FEV1 Among White Individuals in Cohort
eTable 5. Multivariable Regression Model Output for FVC Among Black Individuals in Cohort
eTable 6. Multivariable Regression Model Output for FVC Among White Individuals in Cohort
eTable 7. Comparison of Slopes for Lung Function Trajectory Between Recategorized Groups
eTable 8. Sensitivity Analysis of FEV1 Trajectory Over Time in Patients With Three or More Spirometry Tests
eTable 9. Sensitivity Analysis of FVC Trajectory Over Time in Patients With Three or More Spirometry Tests
eTable 10. Sensitivity Analysis of FEV1 Trajectory Over Time in Patients With Five or More Spirometry Tests
eTable 11. Sensitivity Analysis of FVC Trajectory Over Time in Patients With Five or More Spirometry Tests
eTable 12. Sensitivity Analysis of FEV1 Trajectory Over Time in Patients 30 Years or Older Within Cohort
eTable 13. Sensitivity Analysis of FVC Trajectory Over Time in Patients 30 Years or Older Within Cohort

This supplemental material has been provided by the authors to give readers additional information about their work.

© 2025 Vyas DA et al. *JAMA Network Open*

eFigure 1. Patient Categorizations

Every patient in the cohort was categorized into one of four groups based on categorization of their FEV1 and FVC values as Normal or Abnormal first by the GLI-2012 use of race-specific categories, and then by the GLI-Global, race-neutral categorization. For example, a patient whose FEV1 was categorized as Normal when using the GLI-2012 categorization and Abnormal when using the GLI Global categorization would be identified in the Normal/Abnormal study population.

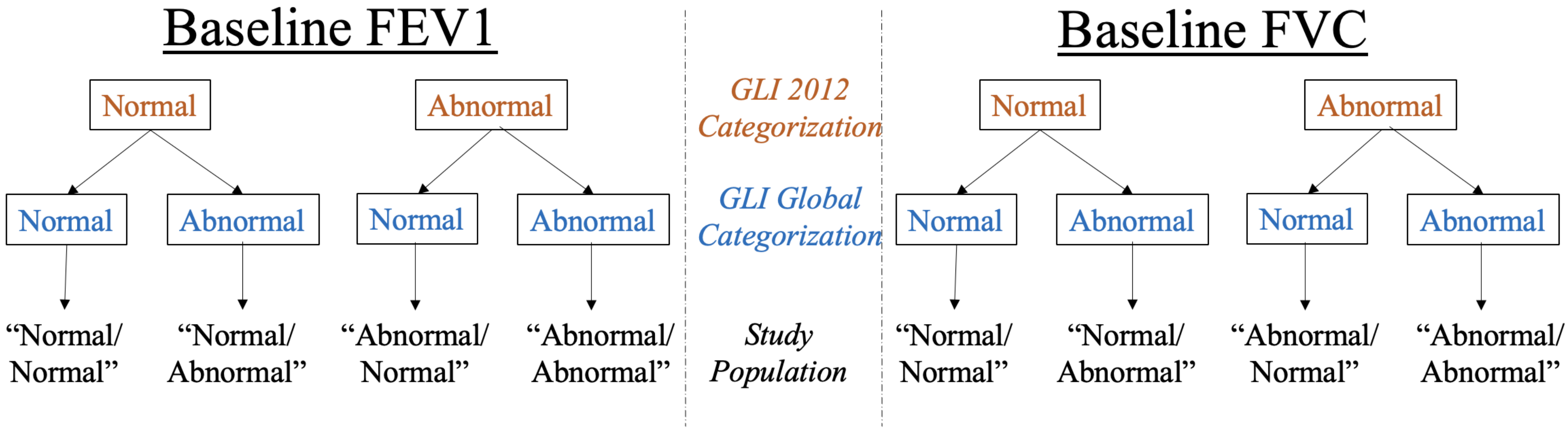

Legend: FEV1 = forced expiratory volume in one second, FVC = forced vital capacity, GLI = Global Lung Initiative

© 2025 Vyas DA et al. *JAMA Network Open*

eFigure 2. Patient Flow Diagram

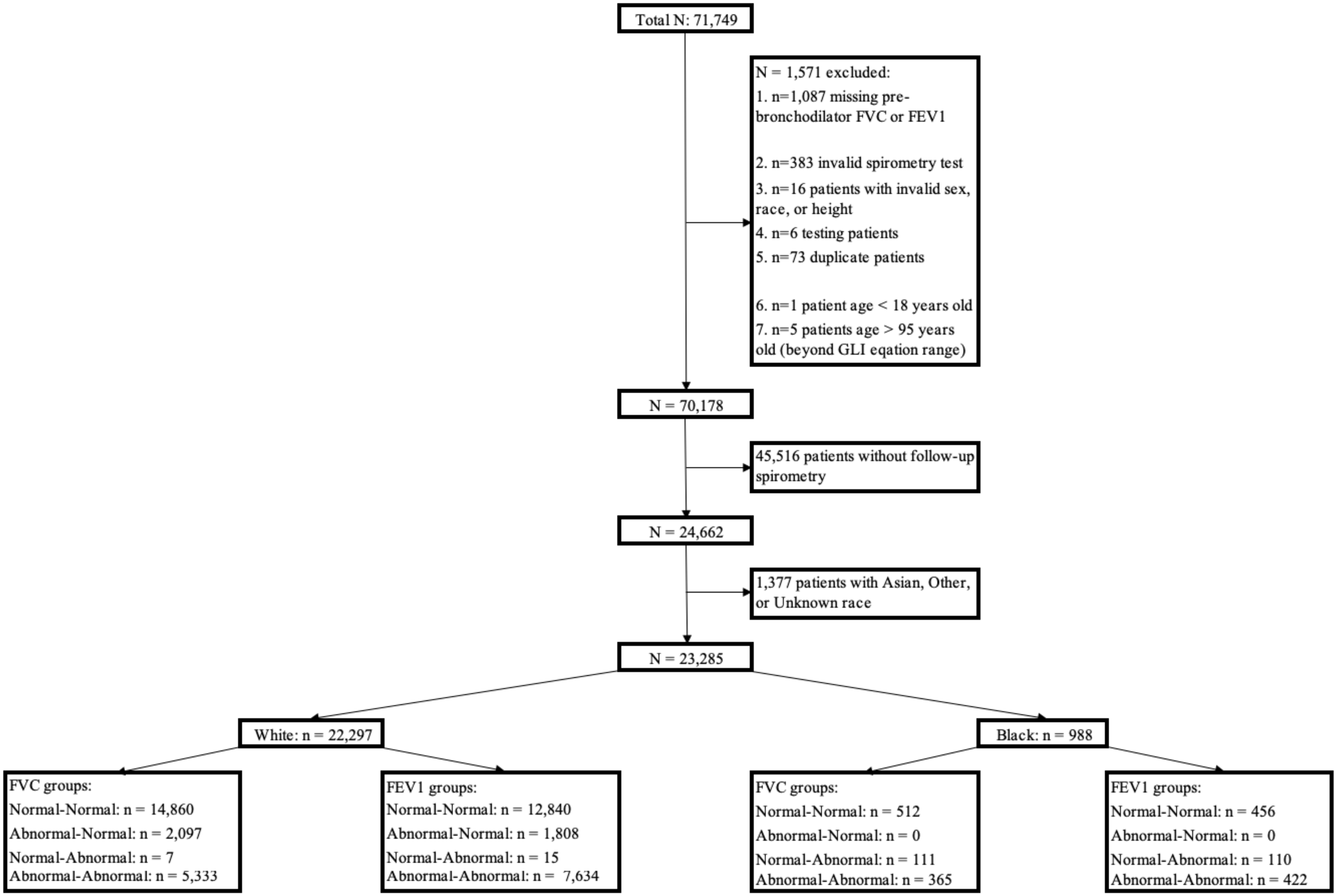

Legend: FEV1 = forced expiratory volume in 1 second, FVC = forced vital capacity, GLI = Global Lung Initiative

© 2025 Vyas DA et al. *JAMA Network Open*

eFigure 3. Follow-up Duration Among Patients With Serial Spirometry in Study Cohort

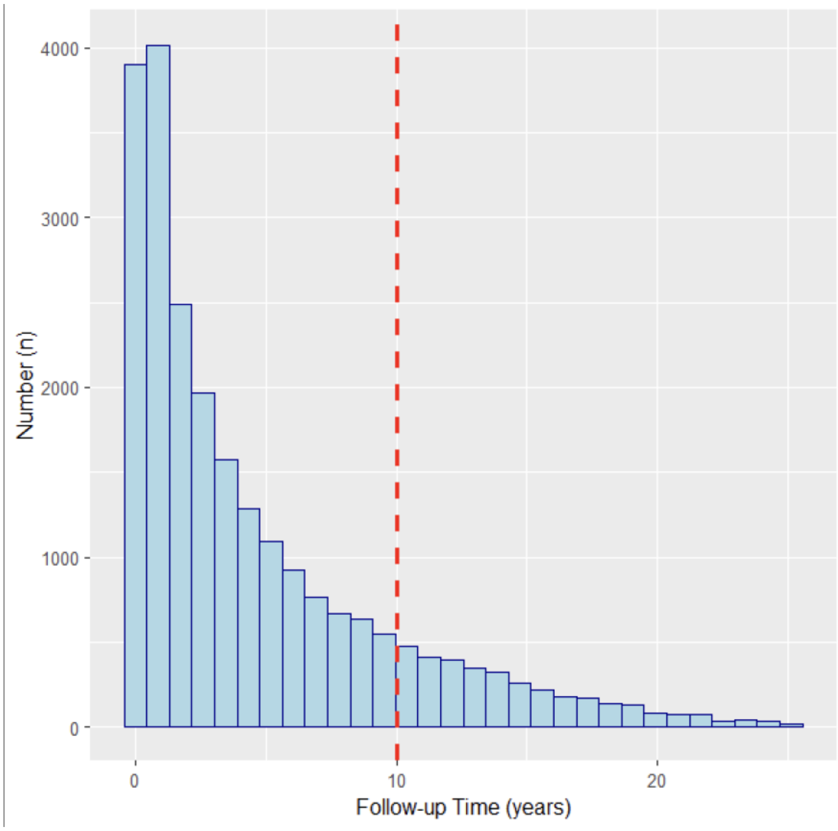

eFigure 4. Model Performance for Multivariable Regression Model for FEV1 Among Black Individuals in Cohort

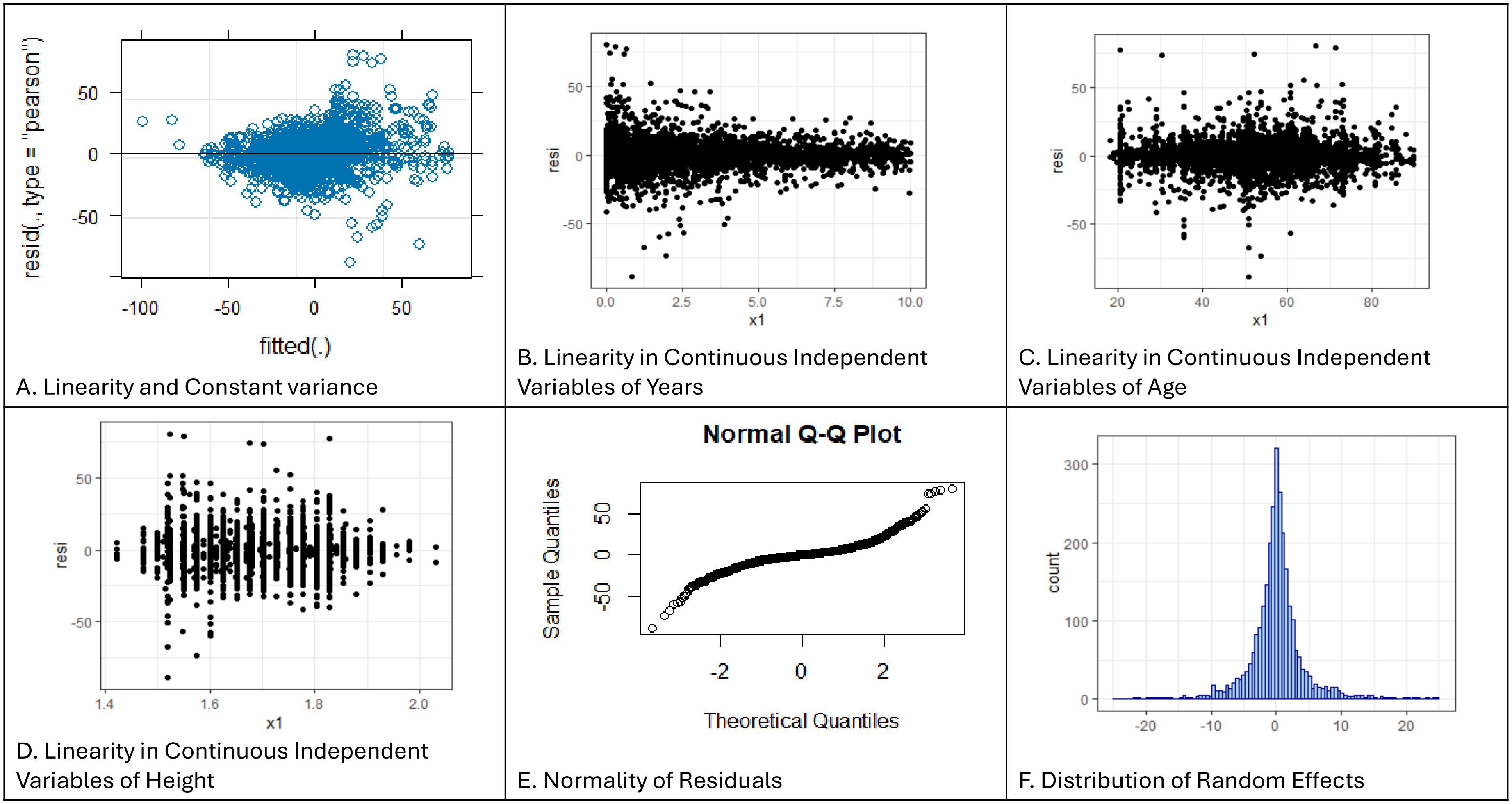

A. Linearity and Constant variance

B. Linearity in Continuous Independent Variables of Years

C. Linearity in Continuous Independent Variables of Age

D. Linearity in Continuous Independent Variables of Height

E. Normality of Residuals

F. Distribution of Random Effects

© 2025 Vyas DA et al. *JAMA Network Open*

eFigure 5. Model Performance for Multivariable Regression Model for FVC Among Black Individuals in Cohort

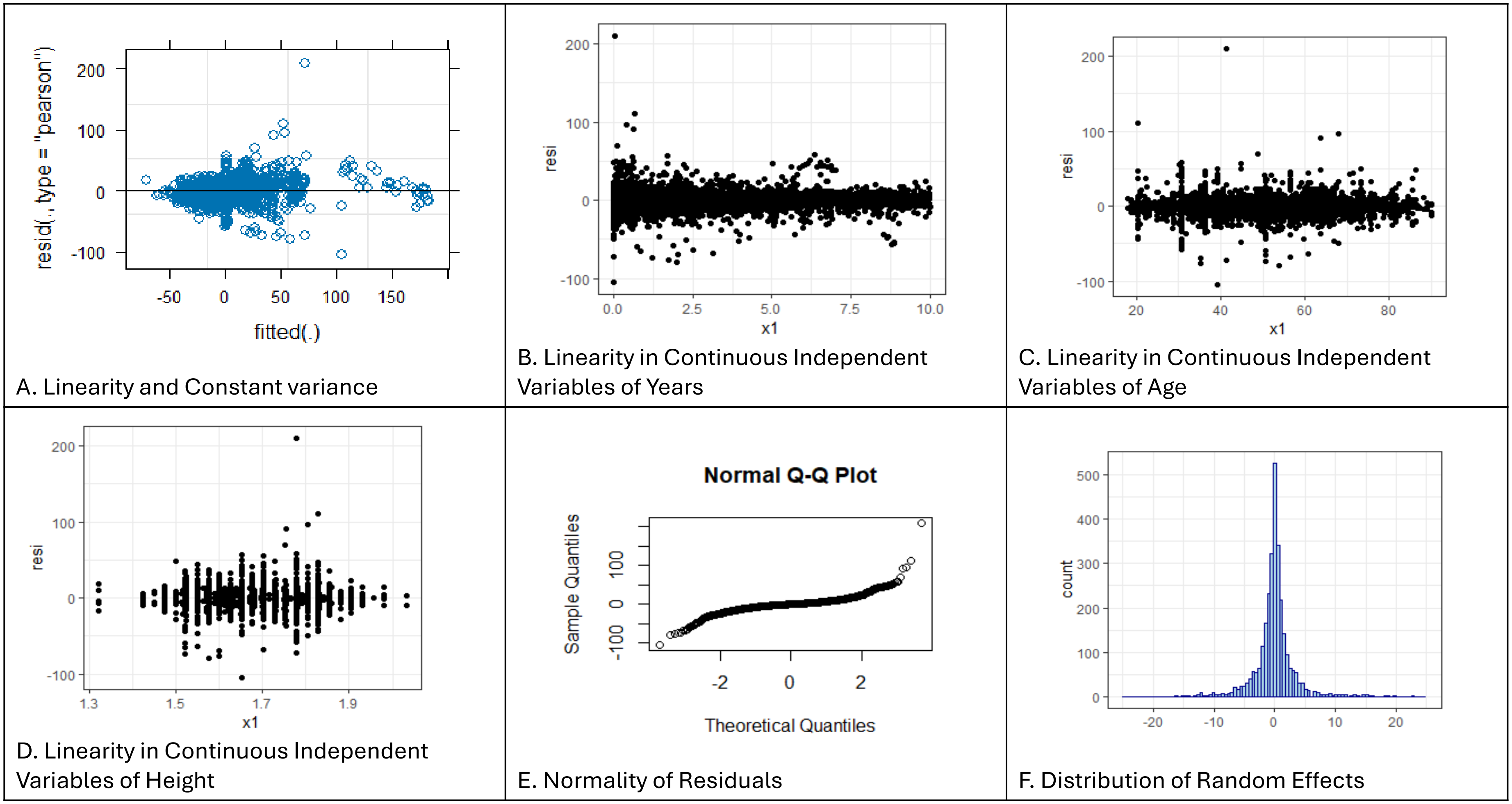

© 2025 Vyas DA et al. *JAMA Network Open*

eFigure 6. Model Performance for Multivariable Regression Model for FEV1 Among White Individuals in Cohort

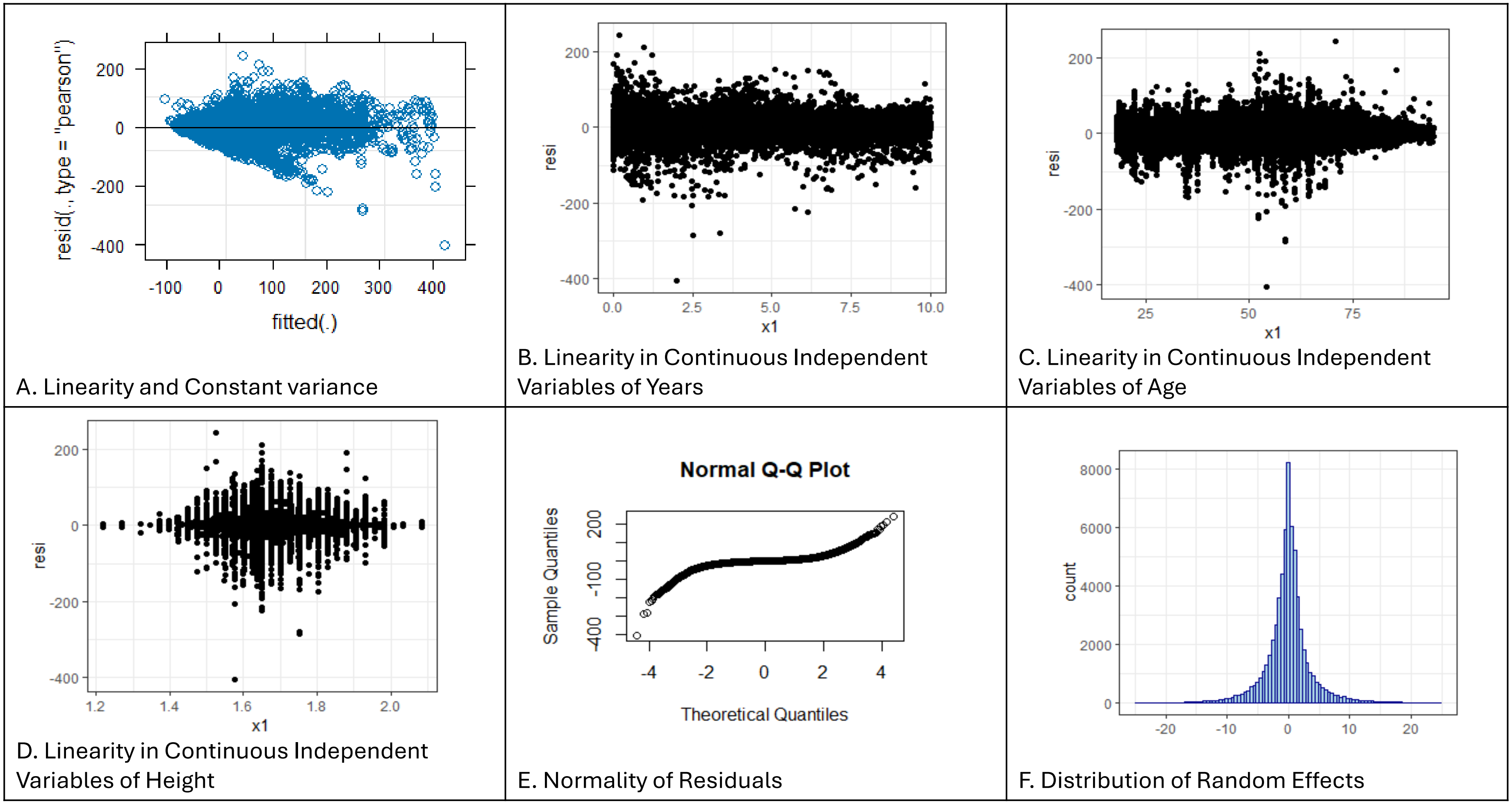

A. Linearity and Constant variance

B. Linearity in Continuous Independent Variables of Years

C. Linearity in Continuous Independent Variables of Age

D. Linearity in Continuous Independent Variables of Height

E. Normality of Residuals

F. Distribution of Random Effects

© 2025 Vyas DA et al. *JAMA Network Open*

eFigure 7. Model Performance for Multivariable Regression Model for FVC Among White Individuals in Cohort

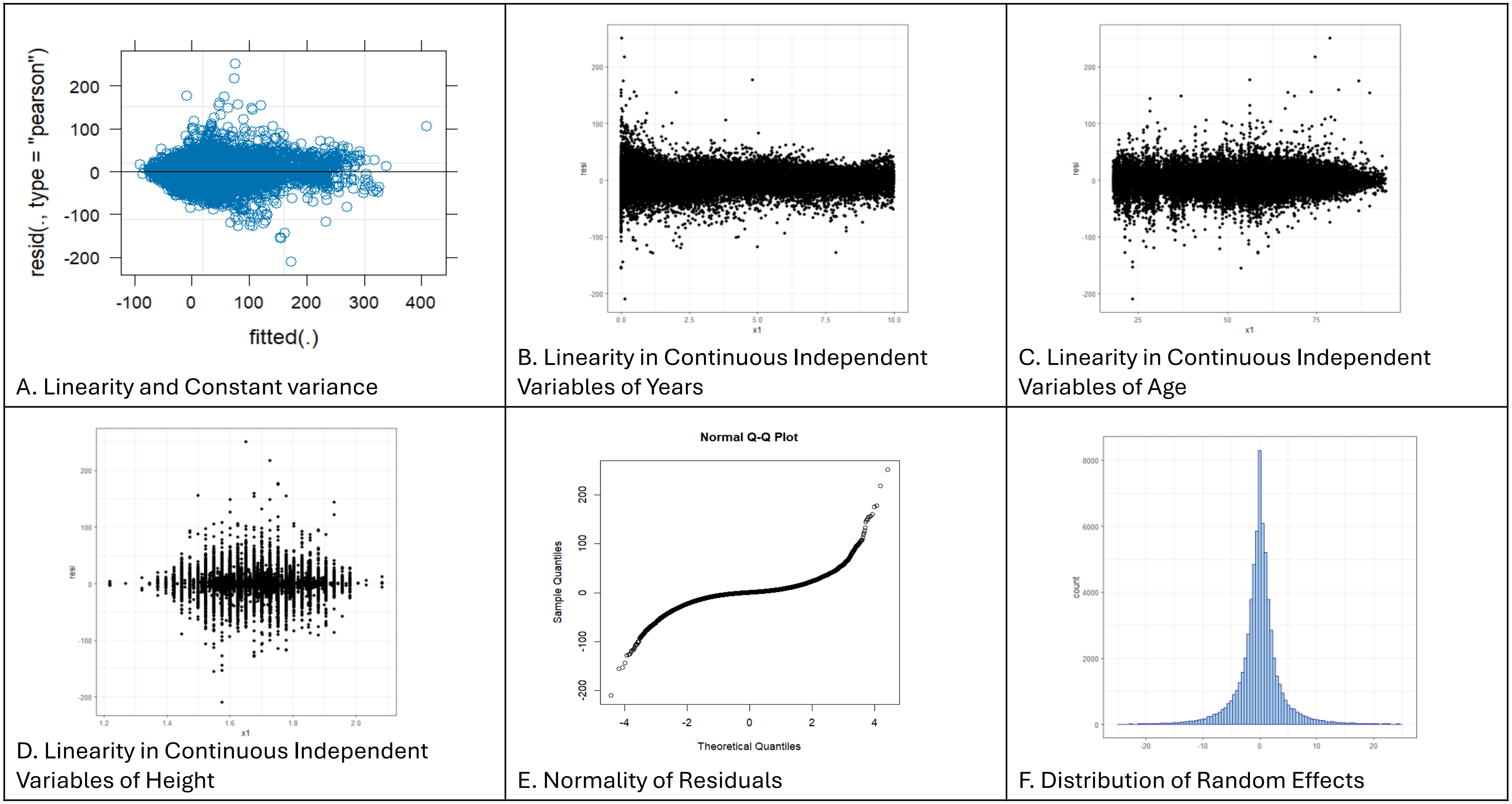

© 2025 Vyas DA et al. *JAMA Network Open*

eTable 1. Number and Time Interval of Spirometry Tests Among Patients in the Analytic Cohort

	Median	25th Percentile	75th Percentile	Minimum	Maximum
Number of tests	3	2	5	2	167
Time interval between tests (months)	4.9	2.0	12.0	0.0	292.6

© 2025 Vyas DA et al. *JAMA Network Open*

eTable 2. Characteristics of White Patients Re-Categorized From Normal to Abnormal Lung Function With GLI-Global Prediction Equation

	Overall (n=22)	By FEV1 Criteria (n=15)	By FVC Criteria (n=7)
Age, years	87.8 (4.3)	87.5 (4.5)	88.6 (4.1)
Age Z score	1.9 (0.3)	1.9 (0.3)	2.0 (0.3)
Male sex	19 (86.4)	12 (80.0)	7 (100.0)
Height, meters	1.7 (0.1)	1.7 (0.1)	1.7 (0.1)
Height Z score	0.6 (0.6)	0.5 (0.6)	0.7 (0.6)
FVC, liters	2.4 (0.4)	2.4 (0.5)	2.5 (0.3)
FEV1, liters	1.6 (0.3)	1.7 (0.3)	1.6 (0.4)

Legend: Mean (SD)
FVC = forced vital capacity, FEV1 = forced expiratory volume in one second

© 2025 Vyas DA et al. *JAMA Network Open*

eTable 3. Multivariable Regression Model Output for FEV1 Among Black Individuals in Cohort

Covariates	*Estimate*	*95% Confidence Interval*		*P values*
Age, per year	-0.11	-0.17	-0.05	<0.001
Male sex	-2.24	-4.59	0.10	0.06
Height, cm	12.18	0.14	24.22	0.05
Spirometry recategorization group[1]				
Normal/Abnormal	1.24	-2.05	1.11	<0.001[2]
Abnormal/Abnormal	11.49	-1.29	0.69	
Yearly decline	-1.59	-2.29	-0.89	<0.001
Interaction: Spirometry recategorization group*yearly decline[1]				
Additional yearly decline within Normal/Abnormal subgroup	-0.47	-2.17	4.65	0.77[2]
Additional yearly decline within Abnormal/Abnormal subgroup	-0.30	9.42	13.56	

Legend: FEV1 = forced expiratory volume in 1 second, cm = centimeters
[1]Spirometry recategorization reference group: Normal/Normal
[2] P values derived from likelihood ratio test

© 2025 Vyas DA et al. *JAMA Network Open*

eTable 4. Multivariable Regression Model Output for FEV1 Among White Individuals in Cohort

Covariates	*Estimate*	*95% Confidence Interval*		*P values*
Age, per year	-0.25	-0.26	-0.23	<0.001
Male sex	1.67	0.95	2.38	<0.001
Height, cm	-16.16	-19.81	-12.51	<0.001
Spirometry recategorization group[1]				
Abnormal/Normal	-1.97	-2.26	-1.69	<0.001
Abnormal/Abnormal	18.10	17.49	18.70	<0.001
Yearly decline	-1.97	-2.26	-1.69	<0.001
Interaction: Spirometry recategorization group*yearly decline[1]				
Additional yearly decline within Abnormal/Normal subgroup	0.16	-0.63	0.94	0.07[2]
Additional yearly decline within Abnormal/Abnormal subgroup	-0.48	-0.93	-0.03	

Legend: FEV1 = forced expiratory volume in 1 second, cm = centimeters
[1]Spirometry recategorization reference group: Normal/Normal
[2] P values derived from likelihood ratio test
*Due to the small sample size, the n=15 White individuals who were categorized as Normal when applying the GLI-2012 prediction equation and categorized as Abnormal when applying the GLI-Global prediction equation (the Normal/Abnormal) were removed from the analysis.

eTable 5. Multivariable Regression Model Output for FVC Among Black Individuals in Cohort

Covariates	*Estimate*	*95% Confidence Interval*		*P values*
Age, per year	-0.08	-0.13	-0.04	<0.001
Male sex	-1.95	-3.79	-0.11	0.04
Height, cm	2.31	-7.12	11.75	0.63
Spirometry recategorization group[1]				
Normal/Abnormal	2.18	-0.34	4.71	<0.001
Abnormal/Abnormal	9.73	8.16	11.31	<0.001
Yearly decline	-1.16	-1.78	-0.54	<0.001
Interaction: Spirometry recategorization group*yearly decline[1]				
Additional yearly decline within Normal/Abnormal subgroup	0.08	-1.42	1.58	0.48[2]
Additional yearly decline within Abnormal/Abnormal subgroup	0.58	-0.38	1.54	

Legend: FVC = forced vital capacity, cm = centimeters
[1]Spirometry recategorization reference group: Normal/Normal
[2] P values derived from likelihood ratio test

© 2025 Vyas DA et al. *JAMA Network Open*

eTable 6. Multivariable Regression Model Output for FVC Among White Individuals in Cohort

Covariates	*Estimate*	*95% Confidence Interval*		*P values*
Age, per year	-0.09	-0.10	-0.08	<0.001
Male sex	0.42	0.04	0.81	0.03
Height, cm	-8.56	-10.53	-6.59	<0.001
Spirometry recategorization group[1]				
Abnormal/Normal	1.08	0.55	1.60	<0.001
Abnormal/Abnormal	12.69	12.34	13.03	<0.001
Yearly decline	-1.52	-1.67	-1.36	<0.001
Interaction: Spirometry recategorization group*yearly decline[1]				
Additional yearly decline within Abnormal/Normal subgroup	0.33	-0.09	0.75	<0.001[2]
Additional yearly decline within Abnormal/Abnormal subgroup	0.66	0.36	0.96	

Legend: FVC = forced vital capacity, cm = centimeters
[1]Spirometry recategorization reference group: Normal/Normal
[2] P values derived from likelihood ratio test
*Due to the small sample size, the n=7 White individuals who were categorized as Normal when applying the GLI-2012 prediction equation and categorized as Abnormal when applying the GLI-Global prediction equation (the Normal/Abnormal) were removed from the analysis.

© 2025 Vyas DA et al. *JAMA Network Open*

eTable 7. Comparison of Slopes for Lung Function Trajectory Between Recategorized Groups

Lung function outcome	Race	Recategorization from GLI-2012 to GLI-Global	Difference in slope compared to reference group	95% CI		P values
FEV1, percent change from baseline	Black	Normal/Normal	Ref	Ref	Ref	Ref
		Normal/Abnormal	-0.47	-2.05	1.11	0.56
		Abnormal/Abnormal	-0.30	-1.29	0.69	0.55
	White	Normal/Normal	Ref	Ref	Ref	Ref
		Abnormal/Normal	0.16	-0.63	0.94	0.70
		Abnormal/Abnormal	-0.48	-0.93	-0.03	0.04
FVC, percent change from baseline	Black	Normal/Normal	Ref	Ref	Ref	Ref
		Normal/Abnormal	0.08	-1.42	1.58	0.91
		Abnormal/Abnormal	0.58	-0.38	1.54	0.23
	White	Normal/Normal	Ref	Ref	Ref	Ref
		Abnormal/Normal	0.33	-0.09	0.75	0.13
		Abnormal/Abnormal	0.66	0.36	0.96	<0.001

Legend: FVC = forced vital capacity, FEV1 = forced expiratory volume in one second, GLI = Global Lung Initiative, CI = confidence interval

© 2025 Vyas DA et al. *JAMA Network Open*

eTable 8. Sensitivity Analysis of FEV1 Trajectory Over Time in Patients With Three or More Spirometry Tests

Race	Group	Yearly FEV1, percent change from baseline	95% CI		P-value
Black, n=573	Normal/Normal (n=234)	-1.66	-2.50	-0.82	Ref
	Normal/Abnormal (n=63)	-1.94	-3.63	-0.25	0.77
	Abnormal/Abnormal (n=276)	-2.06	-2.83	-1.28	0.50
White, n=12,579*	Normal/Normal (n=6,885)	-1.95	-2.32	-1.59	Ref
	Abnormal/Normal (n=1,037)	-1.73	-2.63	-0.82	0.65
	Abnormal/Abnormal (n=4,648)	-2.94	-3.35	-2.52	<0.001

Legend: FEV1 = forced expiratory volume in one second, CI = confidence interval

*Due to the small sample size, the n=9 White individuals who were categorized as Normal when applying the GLI-2012 prediction equation and categorized as Abnormal when applying the GLI-Global prediction equation (the Normal/Abnormal) were removed from the analysis.

eTable 9. Sensitivity Analysis of FVC Trajectory Over Time in Patients With Three or More Spirometry Tests

Race	Group	Yearly FVC, percent change from baseline	95% CI		P-value
Black, n=573	Normal/Normal (n=279)	-1.12	-1.85	-0.38	Ref
	Normal/Abnormal (n=63)	-1.42	-3.12	0.28	0.75
	Abnormal/Abnormal (n=231)	-0.57	-1.39	0.26	0.33
White, n=12,579*	Normal/Normal (n=8,100)	-1.49	-1.69	-1.30	Ref
	Abnormal/Normal (n=1,275)	-1.22	-1.69	-0.76	0.30
	Abnormal/Abnormal (n=3,200)	-1.42	-1.72	-1.12	0.68

Legend: FVC = forced vital capacity, CI = confidence interval

*Due to the small sample size, the n=4 White individuals who were categorized as Normal when applying the GLI-2012 prediction equation and categorized as Abnormal when applying the GLI-Global prediction equation (the Normal/Abnormal) were removed from the analysis.

© 2025 Vyas DA et al. *JAMA Network Open*

eTable 10. Sensitivity Analysis of FEV1 Trajectory Over Time in Patients With Five or More Spirometry Tests

Race	Group	Yearly FEV1, percent change from baseline	95% CI		P-value
Black, n=281	Normal/Normal (n=104)	-1.60	-2.73	-0.48	Ref
	Normal/Abnormal (n=29)	-2.21	-4.58	0.16	0.65
	Abnormal/Abnormal (n=148)	-2.56	-3.53	-1.59	0.21
White, n=6222*	Normal/Normal (n=3,119)	-1.94	-2.49	-1.40	Ref
	Abnormal/Normal (n=545)	-1.70	-3.00	-0.40	0.74
	Abnormal/Abnormal (n=2,553)	-3.04	-3.63	-2.46	0.01

Legend: FEV1 = forced expiratory volume in one second, CI = confidence interval

*Due to the small sample size, the n=1 White individual who was categorized as Normal when applying the GLI-2012 prediction equation and categorized as Abnormal when applying the GLI-Global prediction equation (the Normal/Abnormal) was removed from the analysis.

eTable 11. Sensitivity Analysis of FVC Trajectory Over Time in Patients With Five or More Spirometry Tests

Race	Group	Yearly FVC, percent change from baseline	95% CI		P-value
Black, n=281	Normal/Normal (n=130)	-1.10	-2.05	-0.15	Ref
	Normal/Abnormal (n=24)	-1.93	-4.29	0.43	0.52
	Abnormal/Abnormal (n=127)	-0.89	-1.89	0.10	0.77
White, n=6222*	Normal/Normal (n=3,766)	-1.46	-1.75	-1.18	Ref
	Abnormal/Normal (n=694)	-1.25	-1.90	-0.59	0.55
	Abnormal/Abnormal (n=1,761)	-1.70	-2.12	-1.29	0.36

Legend: FVC = forced vital capacity, CI = confidence interval
* Due to the small sample size, the n=1 White individual who was categorized as Normal when applying the GLI-2012 prediction equation and categorized as Abnormal when applying the GLI-Global prediction equation (the Normal/Abnormal) was removed from the analysis.

© 2025 Vyas DA et al. *JAMA Network Open*

eTable 12. Sensitivity Analysis of FEV1 Trajectory Over Time in Patients 30 Years or Older Within Cohort

Race	Group	Yearly FEV1, percent change from baseline	95% CI		P value
Black: n=909	Normal/Normal (n=411)	-1.62	-2.35	-0.89	Ref
	Normal/Abnormal (n=100)	-2.15	-3.66	-0.64	0.54
	Abnormal/Abnormal (n=398)	-1.96	-2.68	-1.25	0.51
White: n=20846*	Normal/Normal (n=11938)	-2.06	-2.32	-1.81	Ref
	Abnormal/Normal (n=1678)	-1.93	-2.59	-1.27	0.71
	Abnormal/Abnormal (n=7215)	-2.35	-2.66	-2.03	0.17

Legend: FEV1 = forced expiratory volume in one second, CI = confidence interval

*Due to the small sample size, the n=7 White individuals who were categorized as Normal when applying the GLI-2012 prediction equation and categorized as Abnormal when applying the GLI-Global prediction equation (the Normal/Abnormal) were removed from the analysis.

© 2025 Vyas DA et al. *JAMA Network Open*

eTable 13. Sensitivity Analysis of FVC Trajectory Over Time in Patients 30 Years or Older Within Cohort

Race	Group	Yearly FVC, percent change from baseline	95% CI		P-value
Black: n=909	Normal/Normal (n=464)	-1.16	-1.79	-0.52	Ref
	Normal/Abnormal (n=101)	-1.34	-2.77	0.09	0.82
	Abnormal/Abnormal (n=344)	-0.66	-1.41	0.08	0.32
White: n=20846*	Normal/Normal (n=13835)	-1.59	-1.73	-1.44	Ref
	Abnormal/Normal (n=1996)	-1.32	-1.69	-0.96	0.19
	Abnormal/Abnormal (n=5008)	-0.81	-1.05	-0.57	<0.001

Legend: FVC = forced vital capacity, CI = confidence interval
*Due to the small sample size, the n=7 White individuals who were categorized as Normal when applying the GLI-2012 prediction equation and categorized as Abnormal when applying the GLI-Global prediction equation (the Normal/Abnormal) were removed from the analysis.

© 2025 Vyas DA et al. *JAMA Network Open*
