# Supplementary material for: Lung Function Trajectory Using Race-Specific vs Race-Neutral Global Lung Function Initiative Coefficients
Source: JAMA Netw Open. 2025 Apr 25;8(4):e257304. doi: 10.1001/jamanetworkopen.2025.7304 (PMC12032560; doi:10.1001/jamanetworkopen.2025.7304)
Supplement: Supplement 2. — Data Sharing Statement [file jamanetwopen-e257304-s002.pdf]

Data Sharing Statement

Vyas. Lung Function Trajectory Using Race-Specific vs Race-Neutral Global Lung Function Initiative Coefficients. *JAMA Netw Open*. Published April 25, 2025. doi:10.1001/jamanetworkopen.2025.7304

Data
Data available: Yes
Data types: Data (not involving human participants)
How to access data: Per IRB will not share individual PFT reports which may be identifying, but will share other data as requested.
When available: With publication

Supporting Documents
Document types: None

Additional Information
Who can access the data: Anyone requesting
Types of analyses: NA
Mechanisms of data availability: NA
